# Turbine Blade Illusion

**DOI:** 10.1177/2041669517710031

**Published:** 2017-05-25

**Authors:** George Mather, Rob Lee

**Affiliations:** School of Psychology, University of Lincoln, Lincoln, UK

**Keywords:** 3D perception, shapes/objects, scene perception, surfaces/materials

## Abstract

In January 2017, a large wind turbine blade was installed temporarily in a city square as a public artwork. At first sight, media photographs of the installation appeared to be fakes – the blade looks like it could not really be part of the scene. Close inspection of the object shows that its paradoxical visual appearance can be attributed to unconscious assumptions about object shape and light source direction.

In January 2017, wind turbine manufacturer Siemens installed one of its B75 turbine blades in Victoria Square, Hull, to mark the start of the city’s year as UK City of Culture. The ‘Blade’ installation (January 8–March 18, 2017) was conceived by artist Nayan Kulkarni. These turbine blades are huge 75-m long fibreglass, balsa wood and resin composite objects, cast as a single element, and are claimed to be the world’s largest handmade components. Nayan Kulkarni declared that the act of installation in the square changed the blade from an industrial component into a readymade artwork, in the tradition started in 1913 by Marcel Duchamp with his ‘readymades’ which included bicycle wheels, shovels, urinals and bottle racks.

Duchamp considered readymades to be an antidote to ‘retinal art’, to emphasise the conceptual qualities of art rather than their purely visual appearance. But the Blade certainly creates a striking *visual* impression, a metaphorical poke in the eye. Some of this impact must come from its massive scale and its lightness and smoothness, with some specularities, so incongruous against the textures and colours of the ornate Victorian architecture of the square. The local newspaper (*Hull Daily Mail*, January 9, 2017) reported some visitor reactions. One commented that “It looks like a big tooth. It looks a bit odd to be honest.”

Art critic Adrian Searle (*The Guardian*, February 5, 2017) declared: “An elegant, lovely piece of engineering, it is somehow diminished by being shorn of its function. It is only big.”

But it is more than big. Something else seems to be at work too, at least to the eyes of vision scientists. At first sight, the initial media photographs of the installation seemed to be clumsy fakes. The blade appears to be a cylindrical object, strangely out-of-keeping with the local environment, lit differently, as though it was superimposed on the scene digitally (see top two photographs in Figure 1), but the blade really was there. So what is going on in the photographs?

**Figure 1.**
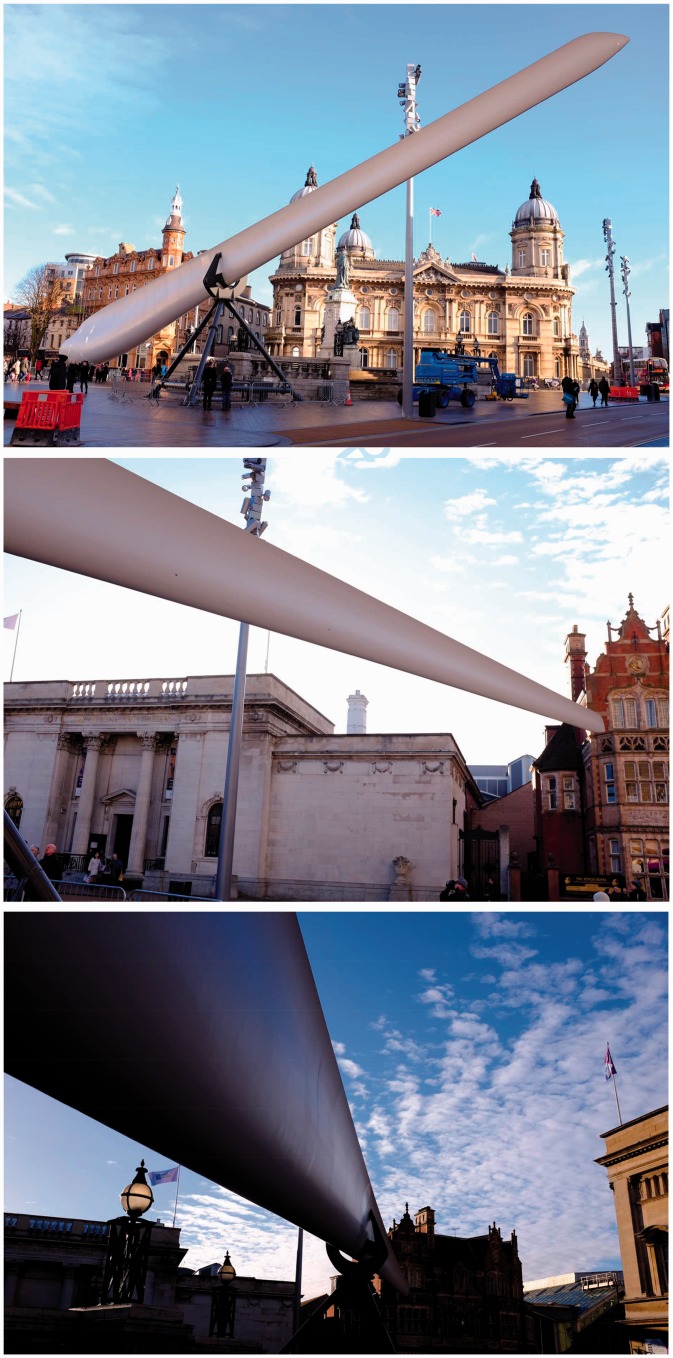
Three photographs of the ‘Blade’ installation in Victoria Square, Hull, UK. In the top two photographs, the blade appears to be cylindrical, and its lighting seems out of place with the daylight illuminating the rest of the scene. In the bottom photograph, the actual S-shaped profile of the blade is more apparent.

Closer inspection on-site (bottom photograph in Figure 1) reveals that one side of the blade is indeed convex and approximately cylindrical. But the profile of the other side is more complex, with both a convexity and a concavity (as shown in Figure 2). The blade’s cross-section is that of a wing, unsurprising given that its function is to generate lift which causes the turbine to spin. The blade’s leading edge is rounded while its trailing edge tapers to a sharp point. The blade had been installed in the square with its leading edge facing the ground and the sharper trailing edge pointing skyward. The photographs actually show the side which is both convex and concave (right side in Figure 2); yet, the entire face appears to be convex, and this seems to be the source of the blade’s paradoxical appearance.

**Figure 2.**
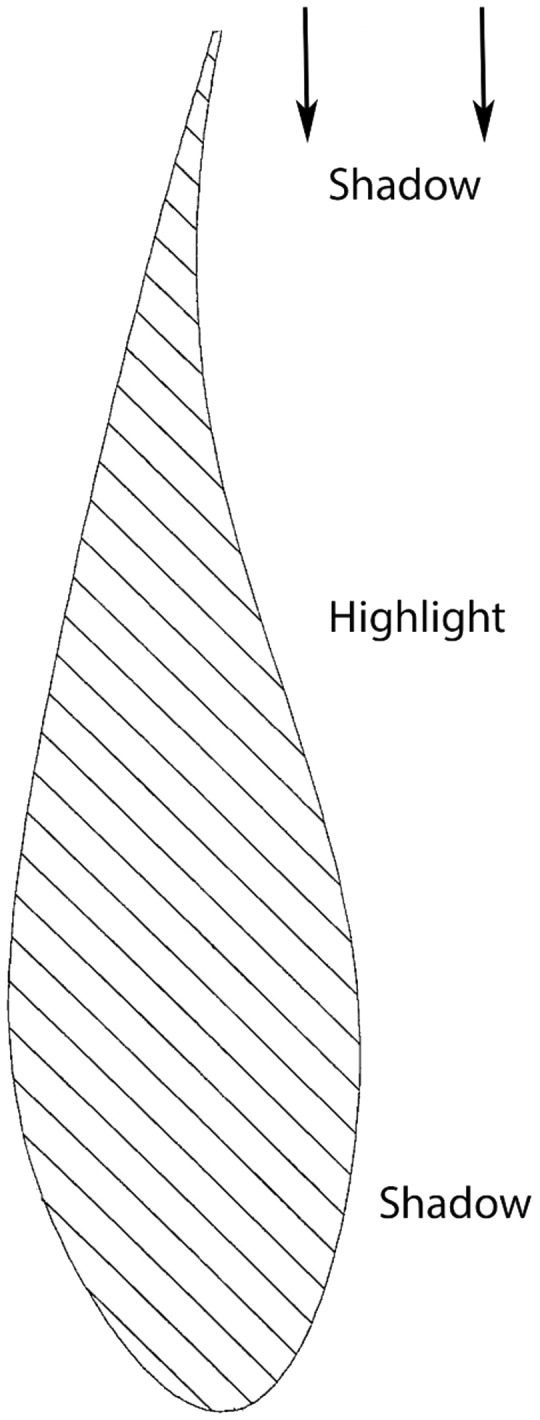
Diagram illustrating the cross-section of a turbine blade such as that shown in Figure 1. The cross-section is that of a wing, with a rounded leading edge (bottom) and a trailing edge which tapers down slightly to impart lift (top). The photographs in Figure 1 show the righthand side of the blade as depicted in the cross-section.

When illuminated by overhead daylight, the lower part of the blade, close to the rounded leading edge is in shadow. The upper part of blade below the trailing edge is also in shadow due to the concave profile. The middle part of the blade receives daylight and appears lighter. The pattern of light and shade across the side of the blade is ambiguous of course. It could be caused by a convex surface, or a more complex combination of concave and convex curvature. The surface curvature we perceive relies on unconscious assumptions or ‘priors’ about natural visual objects and scenes, which are integrated with incoming sensory data (Kersten, Mamassian, & Yuille, 2004). Two priors seem to be operating in the scene. The first assumption is that objects tend to be convex rather than concave (Langer & Bülthoff, 2001), so the local evidence provided by the blade’s shading is interpreted in terms of a cylindrical shape lit from the front (Kleffner & Ramachandran, 1998), and specular reflections reinforce this interpretation (Adams & Elder, 2014). The second assumption is that the most likely direction of the global light source is overhead (Mamassian & Goutcher, 2001). So there is an inconsistency between the blade and the rest of the scene in terms of apparent light source direction, subtly reinforcing the visual impression that the blade is out-of-place.

As a test of this account of the blade’s appearance, we created virtual versions of the convex (C-shaped) profile and the more complex (S-shaped) profiles, lit either from above or from the front. Figure 3 shows these two profiles. The left-hand images depict the C-shape; the right-hand images depict the S-shape. The top pair of images is lit from above and the bottom pair of images is lit from the front. Notice that, as expected on the basis of the blade’s appearance, the bottom-left (C-shape) and top-right (S-shape) images appear similarly cylindrical and lit from the front, though the former is lit from the front and the latter is lit from above. The inset figures show oblique views, which are included in fly-around animations in supplementary materials. Marlow and Anderson (2016) recently investigated ambiguities in the apparent shape of 3D surfaces defined solely by luminance gradients, and showed that the addition of motion or texture information can disambiguate surface shapes similar to those we used. So the illusion we describe depends on keeping the blade very clean.

**Figure 3.**
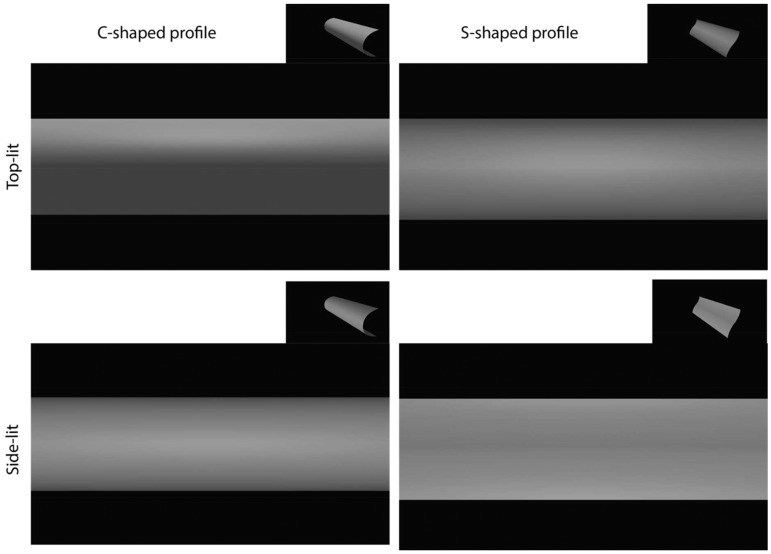
Synthetic images created using Blender™, showing a C-shaped object (left) and S-shaped object (right) illuminated either from above (upper row) or from the front (lower row). The lightness profile of the front-lit C-shaped object (bottom left) is similar to that of the top-lit S-shaped object. Both objects appear to be cylindrical, approximately reproducing the appearance of the blade in the photographs of Figure 1.
